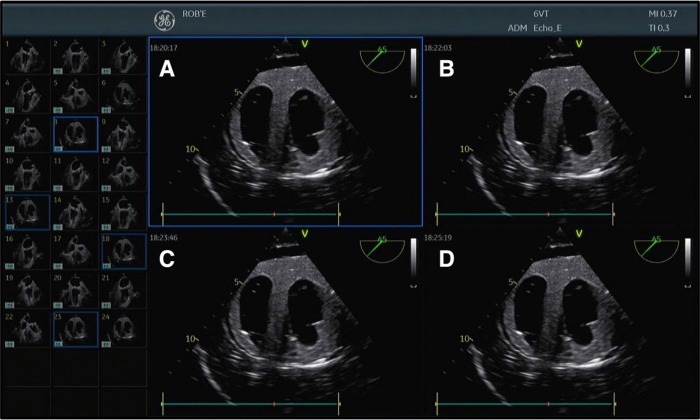# Concept, Design, and Preclinical Testing of a Remote-Control Robotic System for Transesophageal Echocardiography

**DOI:** 10.1016/j.shj.2024.100352

**Published:** 2024-08-26

**Authors:** Jury Schewel, Ryan D. Madder, Dimitry Schewel

**Affiliations:** a Marienkrankenhaus Hamburg gGmbH, Department for Cardiology, Angiology, and Intensive Care, Hamburg, Germany; b ROB’E GmbH, Hamburg, Germany; c Frederik Meijer Heart & Vascular Institute, Grand Rapids, Michigan, USA

**Keywords:** Artificial intelligence, Automation, Diagnostics, Echocardiography, Echocardiographic guidance, Guiding, Heart valve disease, Imaging, Intraprocedural imaging, Radiation, Robotic, Screening, Structural heart interventions, Ultrasound

## Abstract

**Background:**

Interventional echocardiography (IE) plays a critical role in guiding structural heart interventions. IE specialists face challenges including high radiation exposure and unfavorable ergonomics. To address these issues, a novel remote-control robotic (RCR) system for transesophageal echocardiography (TEE) control has been developed. This study aims to describe the novel RCR system and to assess its performance in bench tests and in vitro models in terms of functionality, image quality, and reproducibility.

**Methods:**

Bench testing and in vitro testing were performed using the RCR system. All tests were performed using the GE 6VT-D TEE probe and the GE Vivid E95.

**Results:**

Key findings include proof of concept through bench testing, remote control of all five degrees of freedom of the TEE probe, and reliable, fast, and accurate reproducibility using automated navigation. The ROB’E Base is securely attached to the operating table, optimizing the footprint in the operating room. The ROB’E Guide accurately performs the forward and backward motion of the flexible portion of the TEE probe, stabilizing the achieved positions and preventing twisting during rotation. The ROB'E RCR system can store and reproduce TEE probe positions and has demonstrated reliable and accurate automated reproducibility in both bench and in vitro tests.

**Conclusions:**

The ROB'E RCR system for TEE overcomes the limitations of conventional IE by using a RCR approach that eliminates the need for the echocardiographer to be physically present in the operating room. Thus, it significantly reduces radiation exposure and demonstrates its capabilities to improve image quality, reproducibility, and overall safety in IE.

## Highlights

• Developed a novel remote-control robotic system for transesophageal echocardiography (TEE), addressing key challenges in interventional echocardiography.
• The robotic echocardiography system reduces radiation exposure for echocardiographers and enhances procedural safety by enabling remote control of the TEE probe.
• The robotic echocardiography system demonstrated reliable and accurate reproducibility of TEE probe positions through automated navigation.

## Introduction

The field of structural heart interventions has experienced significant growth in recent years, driven by advances in device technology and procedural techniques. As a result of this development, transcatheter edge-to-edge repair (TEER) for mitral valve regurgitation, transcatheter aortic valve replacement, and left atrial appendage occlusion have been incorporated into international guidelines. Similarly, the results of TEER for the treatment of tricuspid regurgitation show promise. The variety of such therapeutic options is growing exponentially every year, with new technologies such as transcatheter mitral valve replacement, tricuspid valve replacement, and interatrial shunt implantation for heart failure.^1^ These therapies share a common need for visualization of intracardiac structures and therapeutic devices. In addition to fluoroscopy, interventional cardiology uses transesophageal echocardiography (TEE) for both preprocedural diagnostics and intraprocedural guidance of such therapies. Thus, the interventional echocardiographer (IE) is an integral part of the heart team, mandatory for decision-making, guiding, and monitoring such complex interventional cardiac procedures. However, it has taken more than a decade for the indispensable role of the IE to be recognized and properly valued.^2^^,^^3^ This is reflected in the development of standardized recommendations for the training of IEs by the American Society of Echocardiography^4^^,^^5^ and the European Association of Cardiovascular Imaging.^6^ These curricula are intended to ensure qualified training to ensure a high level of safety, quality, and effectiveness of interventional therapies for structural heart disease.

The IE approach presents significant challenges by requiring the echocardiographer to be physically present in the operating room (OR), manually steering the TEE probe, and simultaneously controlling the echocardiography machine. While interventional procedures are usually accompanied by fluoroscopic imaging, the TEE operator is necessarily required to stand close to the source of radiation. Thus, studies have shown that the radiation exposure of IE specialists is 10 times higher than that of interventional cardiologists.^7^, ^8^, ^9^, ^10^, ^11^ The exposure to radiation requires IE to wear lead aprons, which may place them at risk for orthopedic injuries similar to those commonplace among interventional cardiologists.^12^, ^13^, ^14^ In addition, echocardiographers performing IE face a variety of procedural challenges, including the difficult adjustment and repetitive reproduction of high-quality echo images and the avoidance of esophageal and gastric injury.^15^^,^^16^ These challenges and limitations, along with the rapidly increasing number of procedures, have led to a shortage of qualified echocardiographers who are able or willing to perform IE procedures, posing a major obstacle to the treatment of structural heart disease.^17^

In response to these challenges, a novel robotic system for manipulating a TEE probe has recently been developed.^18^, ^19^, ^20^ The present work aims to present the concept, design, and results of preclinical testing of this novel cooperating robot (cobot) system developed by the ROB’E GmbH (Hamburg, Germany) that enables remote-control robotics (RCRs) for TEE as a potential solution to address the mentioned limitations and challenges of IE. The specific objectives of the present work are:

1. To describe the novel RCR system,
2. To evaluate the performance of the RCR TEE system in terms of maneuverability and handling of the controller.
3. To assess the performance of the RCR TEE system in an in vitro model, especially focusing on the evaluation of the quality of image reproducibility.

## Methods

### Concept and Design

**Figure 1:**
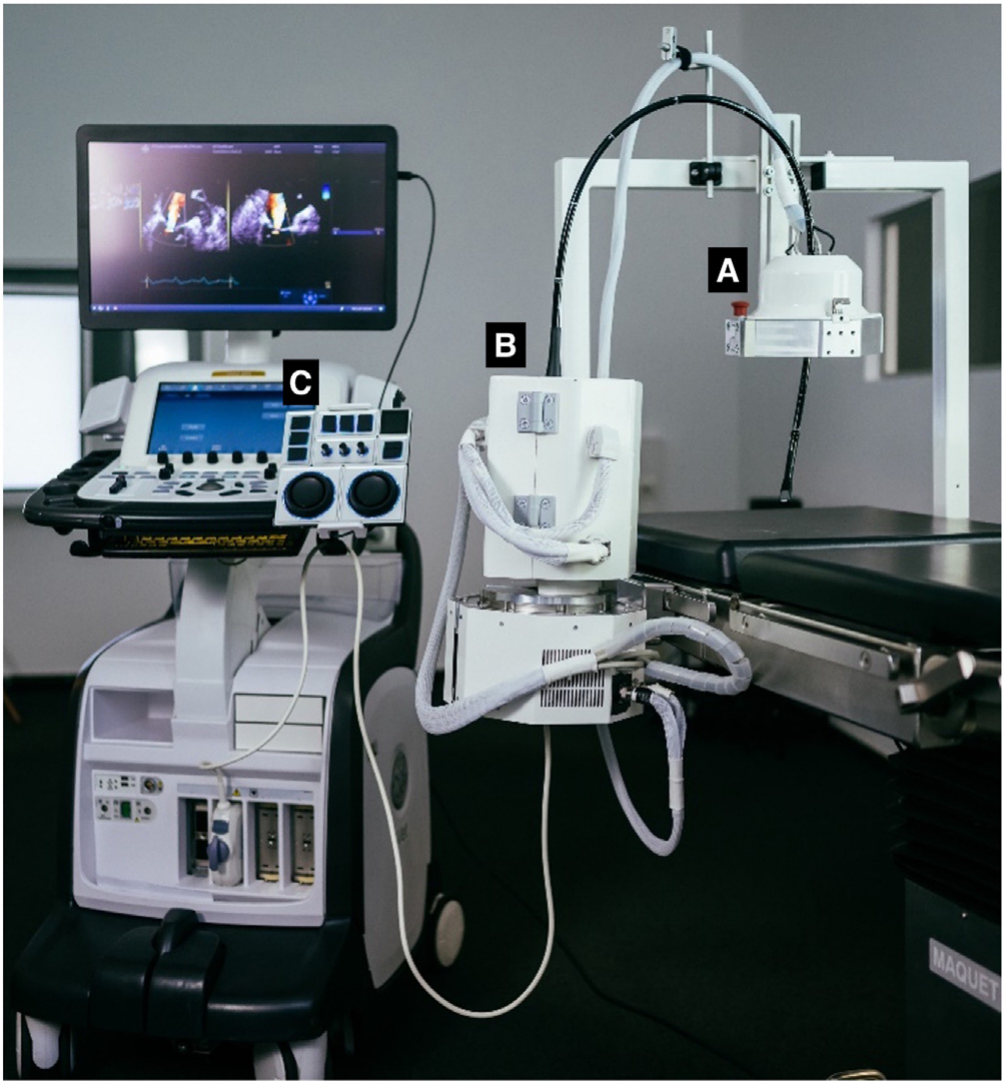
Conceptual design and setup of the ROB’E RCR system for TEE consisting of three parts: (a) ROB’E Guide; (b) ROB’E Base with the integrated ROB’E Computer; and (c) ROB’E Controller Abbreviations: RCR, remote-control robotic; TEE, transesophageal echocardiography.

The RCR system (ROB'E GmbH, Hamburg, Germany) for TEE was developed to address the challenges associated with conventional IE by providing a co-robotic remote control for TEE probes that allows remote control of all five degrees of freedom (DOF), as shown by Hahn et al.^21^ The system is designed to be compatible with any TEE probe currently available on the market. It consists of the following parts (Figure 1).

- RCR “ROB’E” Guide: This component is located near the patient's mouth and comprises the flexible portion of the TEE probe that is inserted into the patient's esophagus. It is responsible for advancing and retracting the TEE probe, stabilizing the position reached, and rotating the probe around its longitudinal axis. Force limiters are incooperated, as well as a stop button to relax all motors immediately.
- RCR “ROB’E” Base: This component serves as the foundation of the RCR system and is placed on any side of the operating table at the level of the patient's head near the RCR ROB’E Guide.
It houses the motors and actuators necessary to operate four of the five DOFs of the probe. It allows axial rotation of the probe handle, operation of the large and small wheels that control antegrade-retrograde and right-left bending, and actuation of the buttons that control the direction of the probe's scan plane. The maximum movement of the TEE probe that can be realized by the ROB'E Base is limited by the probe itself, providing sufficient safety. Force limiters are also incorporated to prevent erroneous movements of the TEE probe.
- RCR “ROB’E” Controller: The controller serves as the user interface for interacting with the RCR Guide and Base. The controller is connected to the ROB'E Base via a detachable cable system. This setup ensures reliable communication and control while allowing for easy disconnection if needed. It allows the IE to precisely manipulate all aspects of the TEE probe. Moreover, it provides functionalities for saving multiple probe positions, thereby enabling quick navigation and reproduction of desired target views.
- RCR “ROB’E" Computer: An integrated computer is responsible for processing user input from the controller and sending commands to the ROB’E Guide and ROB’E Base. It ensures seamless communication between the user and the robotic system.

### RCR “ROB’E” Workflow

First, a physician or sonographer manually inserts the distal end of the TEE probe into the patient's mouth and further into the esophagus. Next, the flexible portion of the TEE probe is clamped into the ROB’E Guide, located directly in front of the patient's mouth. Finally, the handle of the TEE probe is inserted into the ROB’E Base and locked in place.

The IE can then move to the echo machine and distance themselves away from the operating table and the radiation source. Using the RCR Controller, the IE can precisely and individually control the TEE probe to navigate to any desired position or view (Supplemental Video 1-4). Preset “Quick Views” allow the physician to automatically navigate to the most-used echo views and then fine-tune them manually by using the controller (Video 5). When the physician is satisfied with the image achieved, the position can be stored to reproduce this view later, as often as necessary.

### Test Setups

#### Echo Machine

The ROB'E RCR system can be used with any commercially available TEE probe, regardless of vendor, by using appropriate adapters. For the purposes of this study, the system was adapted to the 6VT-D TEE probe (GE HealthCare), and all tests were performed with a commercially available GE Vivid E95.

#### Bench Testing

The bench tests were performed in a test setup in which the RCR system was mounted on the rail of a mock operating table. The TEE probe was clamped, and the distal, flexible section, which would typically be positioned in a patient’s esophagus, was left free to hang. All DOF were then repeatedly activated and manipulated using the controller, allowing different positions to be approached manually and automatically.

#### In Vitro Testing

For the in vitro test, a nonbeating heart model (Blue Phantom, CAE Healthcare Inc) was used. This simulator, developed for TEE training, consists of a silicone block with good acoustic properties, into which a passive, nonbeating silicone heart model is incorporated. The silicone block contains a channel that simulates the esophagus and allows anatomically correct acquisition of transesophageal and transgastric views. After insertion of the distal end of the flexible part of the TEE probe into the model, different clinically relevant target views (mid-esophageal 4-chamber view, bicommissural view, 3-chamber view, short axis view, and trans-gastric short axis view) were first acquired robotically by manipulating the TEE probe using the ROB’E Controller. The coordinates of each achieved target view were stored by the robotic device. When all target views were reached and stored, multiple attempts were made to automatically reproduce the target views in series by pushing the relevant “stored view” button on the robotic device. The coordinates of the views reached using these “stored view” buttons were compared with the saved coordinates of the target position, and the echo images reached were similarly compared with the previously saved ones (Video 5).

## Results

### Bench Testing

After inserting the TEE probe into the ROB’E Guide and Base, all subsequent manipulations of the TEE probe were performed using the RCR Controller. Using this setup, all motors were driven accurately, and each DOF of the TEE probe could be manipulated with high precision. By recording the motor’s coordinates, different positions could be stored and reproduced multiple times. The motor coordinates of the initially stored positions and the repeatedly reproduced positions were compared. No significant differences were documented.

### In Vitro Testing

Following successful bench tests, the RCR system was further evaluated using a static, nonbeating heart simulator (Blue Phantom, CAE Healthcare Inc). Multiple operators manipulated the TEE probe using the RCR Controller to evaluate the system's precision and control over the DOF of the probe. The ROB’E RCR system successfully performed all desired manipulations, demonstrating its ability to replicate the motions of a conventional TEE procedure.

**Figure 2:**
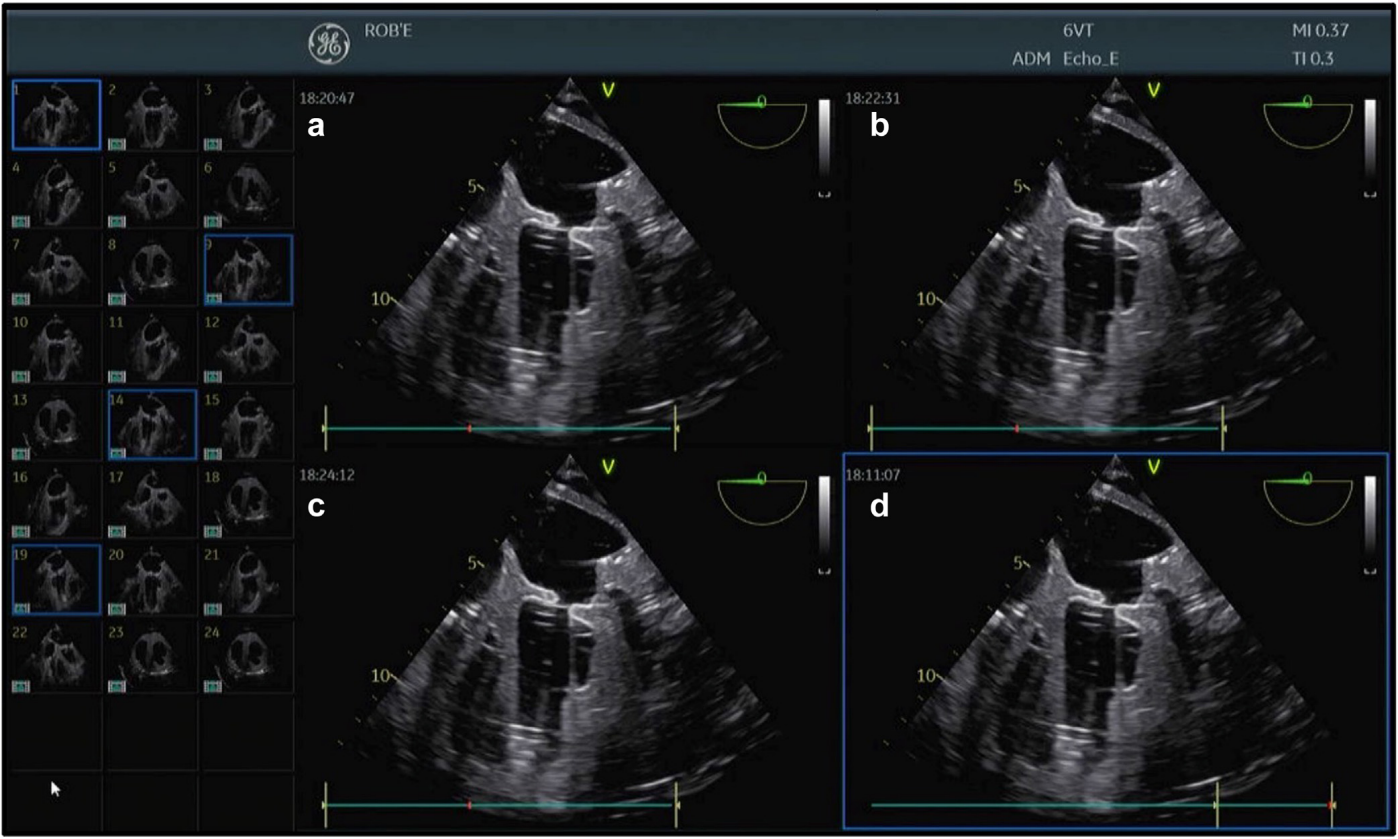
Demonstration of the accurate reproducibility of the ROB’E RCR system in combination with the GE Vivid E95 and 6VT-D TEE probe in a nonbeating heart model (Blue Phantom). (a) First reached 4-chamber view; (b) first time reproduced 4-chamber view after multiple other views; (c) second time reproduced 4-chamber view after multiple other views; (d) third time reproduced 4-chamber view after multiple other views Abbreviations: RCR, remote-control robotic; TEE, transesophageal echocardiography.

**Suppl_Figure 1:**
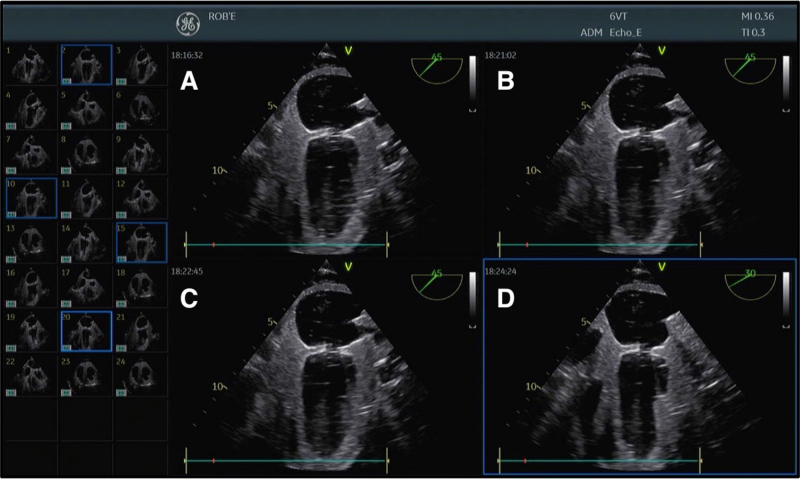


**Suppl_Figure 2:**
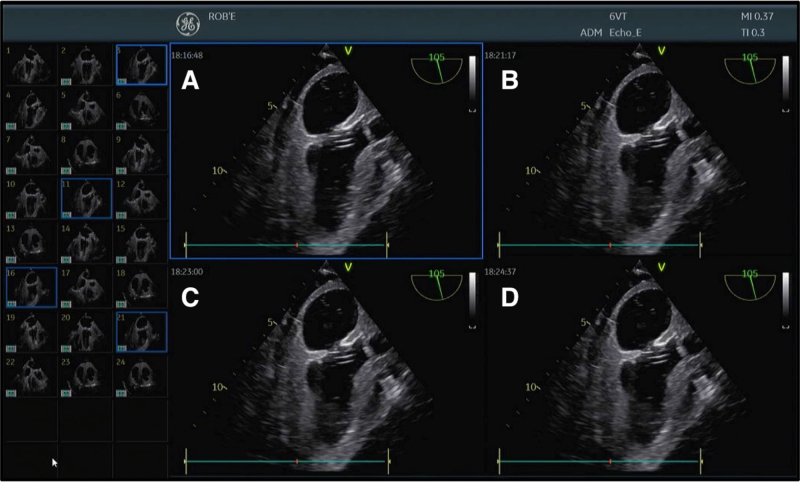


**Suppl_Figure 3:**
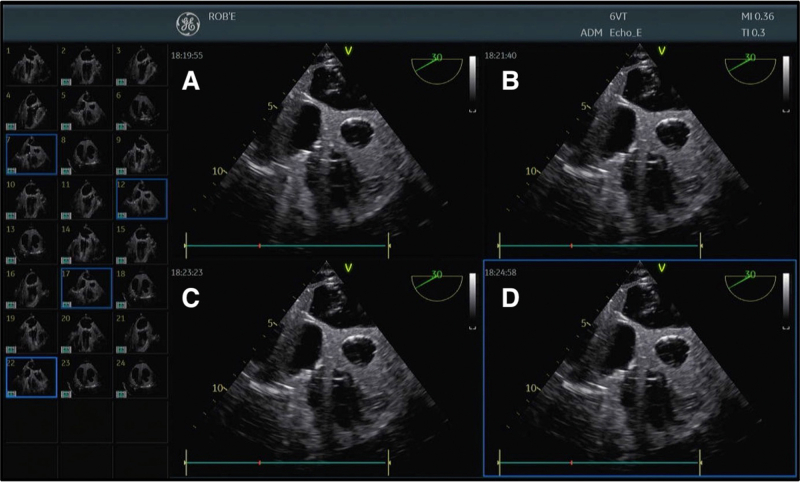


Echocardiographic images were obtained throughout the in vitro testing, confirming the system's potential to visualize cardiac structures. The static heart simulator allowed a detailed assessment of image quality and verified that the remote-controlled TEE probe provided images comparable to those obtained by conventional TEE. Different views were targeted: 4-chamber, 2-chamber, 3-chamber, short-axis, and transgastric views. In addition, the TEE probe positions could be stored by the RCR system and accurately reproduced multiple times (Figure 2, Supplement Figures 1-4). The reproduced images were comparable to the stored images. No unintended movements or malfunctions were observed during the tests, demonstrating the system's reliability.

## Discussion

The development of an RCR for TEE offers a promising solution to address the challenges and limitations associated with manual TEE procedures, particularly in the context of structural heart interventions. In the present study, the novel RCR system for TEE was introduced, and the in vitro experience was described. The main results of the present work are:

1. The proof of concept of the described ROB’E RCR system was demonstrated in series bench testing.
2. The ROB’E RCR system showed good compatibility with the GE 6VT-D TEE probe and the GE Vivid E95 echo machine.
3. All five DOFs of the TEE probe could be easily and accurately controlled remotely by operating the ROB’E RCR system.
4. By utilizing the automated navigation of the ROB’E RCR system, reliable, fast, and accurate reproducibility of different positions and corresponding echo views could be documented both in benchtop and in vitro tests.

### RCR Systems for Transesophageal Echocardiography

For several years, many research groups have made developments in remote robotic ultrasound systems. Especially since the COVID-19 pandemic, this area has gained progressive interest in development. In cardiac ultrasound, the focus is mainly on transthoracic echocardiography (TTE), as this diagnostic tool is the most frequently used modality in cardiac care, and the opportunity to have a standardized TTE exam in remote and rural areas would revolutionize how diseases are diagnosed.^22^ Compared to TTE, robotic developments for TEE are rare. So far, only three different concepts for an RCR system for remote control of a TEE probe have been described.^18^, ^19^, ^20^ All three concepts agree that there must be a main unit that allows manipulation of the TEE probe's handgrip and thus ensures control of four of the five possible DOFs. The RCR system described in the present study also includes such a component, which is referred to as the "ROB’E Base." Compared to the previous systems, the ROB’E Base differs in its mobility and the way it is fixed to the operating table. The ROB’E Base is positioned vertically, which offers a clear advantage in terms of space utilization and leaves a significantly smaller footprint in the OR, so that the movement of all actors, such as anesthesia or nursing staff, is less hindered. The ROB’E Base is fixed in any position at the head side and directly to the operating table, so that it follows any movement of the operating table during the procedure, and thus the position of the TEE probe in the patient remains unaffected. This design ensures stable and constant image acquisition.

To adjust the position of the TEE probe in the patient's esophagus, it is important to be able to manipulate the forward and backward movement of the flexible endoscope part of the TEE probe, which is usually done manually by the echocardiographer. Since the esophagus is a continuously moving muscle and often slowly pulls the TEE probe in or out, changing the set position of the distal end of the TEE probe, stabilization of the achieved position is of utmost importance to preserve the obtained echo image. Therefore, a second subsystem, as described by Pahl et al., is essential.^19^ Without such a subsystem, there is also a high risk that rotation of the probe’s handle around the longitudinal axis will result in twisting between the handle and the distal end of the flexible part, which would lead to inaccurate translation of the initial rotation of the handle and produce unacceptably inaccurate control. The only RCR system described to date that includes such a subsystem is that of Sayahkarajy and Faudzi.^20^ In their concept, the authors described an arm design that features a two-link structure with a soft actuator using cylindrical McKibben muscles and a crossed string belt mechanism that allows the gripper to move. Such a concept seems unnecessarily cumbersome and not yet efficient enough for clinical use, especially regarding the space required in the OR. The subsystem described in this paper, called "ROB’E Guide," like the ROB’E Base described above, is fixed in any position at the head end of the operating table and thus follows any change in the position of the operating table. A built-in motor controls the forward and backward movement of the probe in and out of the esophagus and ensures a stable position during the inactive phase, which guarantees that the image obtained is maintained. When the probe handle is rotated by the ROB’E Base, a second motor integrated in the ROB’E Guide ensures synchronous rotation of the flexible section, which prevents the probe from twisting. In this way, this RCR system achieves uniquely direct axial transduction with stable and more precise imaging.

The third and so far unique feature of the ROB'E RCR system is the possibility to store the achieved positions of the TEE probe corresponding to clinically relevant TEE settings and to reproduce them automated on request with high accuracy and speed. Although this modality is obvious, it has not yet been described or tested by any working group. The current prototype of the RCR system reproduces previously saved echo views based on the corresponding engine positions. This is a technical limitation of the current development and will be realized in the next generation using an artificial intelligence based analysis of the echo images. In addition, the tests were performed in a static model, whereas it would be of interest to perform the tests in a nonstatic human or animal model. However, from a clinical point of view, this functionality is highly relevant to significantly reduce procedure duration, radiation exposure for the patient and the entire medical staff, and duration of anesthesia. The ROB’E RCR system tested in the present study showed reliable and precise automated reproducibility of different echo settings (transesophageal and transgastric) in bench tests.

### Clinical Implication

#### Facing the Challenges in Interventional Echocardiography

The realm of multimodality imaging, particularly echocardiography, is pivotal in diagnosing and managing structural heart diseases. Its role spans the entire spectrum of patient care, from initial diagnosis to guiding interventions like mitral and tricuspid valve repairs and left atrial appendage or patent foramen ovale closures and extending into follow-up care. The subspecialty of interventional echocardiography (IE) has rapidly evolved with the advent of transcatheter treatments, albeit not without facing significant challenges such as complex image acquisition, high radiation exposure, and procedural complications like esophageal bleedings, which can extend intervention times and impact clinician concentration.^2^, ^3^, ^4^, ^5^, ^6^, ^7^, ^8^, ^9^, ^10^, ^11^^,^^15^^,^^16^^,^^23^^,^^24^

Despite the development of standardized training recommendations by leading societies in Europe and the United States to ensure competency in guiding structural heart interventions, there remains a notable shortage of experienced interventional imagers. New advancements in fusion-imaging technologies have been designed to enhance intraprocedural guidance, exemplified by tools like Philips EchoNavigator^25^ and Siemens TrueFusion,^26^ which integrate real-time fluoroscopy with continuous echocardiography. Similarly, GE's fusion of echocardiography with computed tomography scans^27^ aims to streamline procedural complexity. However, the cognitive demand of integrating these modalities poses a significant learning curve and necessitates further research to determine their impact on procedural efficiency, clinical outcomes, radiation safety, contrast dosage, and procedural success.^28^

The introduction of intracardiac echocardiography (ICE) represented a significant technological advancement, especially with the improvement in image quality through 3D-/4D-imaging capabilities.^29^ However, comparative analyses have shown that ICE does not definitively outperform traditional TEE guidance in terms of efficacy and was linked to twice as higher rates of pericardial effusion requiring intervention and higher in-hospital mortality rates.^30^ Further data support TEE's superiority in image quality, procedure, and fluoroscopy time efficiency, as well as in achieving more effective mitral regurgitation reduction compared to 3D ICE in mitral TEER.^31^ These insights highlight the need for further research and multicenter trials to comprehensively assess the potential and limitations of advanced 3D/4D ICE technology. Conclusively, TEE remains the state-of-the-art imaging modality for structural heart procedures, and even more, it is used in the diagnostics and in the follow-ups to reassess the pathologies compared to ICE catheters, which could only be used during interventions.

#### New Era of RCR Systems

There is a huge unmet clinical need to improve echocardiographic guidance during structural heart procedures. The described challenge needs to be solved to have more standardized, high-quality imaging. The described technology is just the first prototype of an RCR system bench-tested in a static model; it is the beginning, a proof-of-concept. Lots of technology improvements must be done until implementation in the clinical setting, like improving the user interface, haptic feedbacks, developing artificial intelligence with probe manipulation based on image and force sensors, and much more. The integration of a RCR system for TEE could address the described challenges by reducing radiation exposure to almost zero, as the imager could distance themselves from the X-ray source. Further, it could enable more accurate and precise probe handling while reducing interobserver and interscan variability. It could also improve image quality and consistency while minimizing manual manipulation of the probe, thereby reducing the risk of procedural complications such as esophageal injuries. By potentially shortening procedural times and decreasing the need for repeat interventions, this technology can offer long-term cost savings for health care systems. Moreover, simplifying the procedural aspects of structural heart interventions and potentially enabling remote operation, these RCR systems could facilitate the delivery of high-quality care to patients who otherwise might lack access to such treatments. Such democratization of access to advanced cardiac imaging will make the benefits universally available.

## Limitations

The present work presents a new concept and the corresponding proof of concept from serial technical tests in a benchtop model and in an in vitro heart simulator. Further systematic testing in beating heart simulators, animal models, and humans is required to make reliable statements about functionality, efficacy, and safety, as well as integration in a clinical setting.

## Conclusions

The ROB’E RCR system for TEE tackles the limitations of conventional IE by employing a RCR approach that eliminates the need for the echocardiographer to be physically present directly at the operation table or even in the OR. Thus, the implementation of such an RCR system would guarantee a reduction of radiation exposure, especially for interventional echocardiographers, from the currently highest level to zero. The preclinical testing of this RCR system has demonstrated its feasibility and effectiveness. They demonstrated that this innovative approach might improve image quality and reproducibility while reducing the risks associated with conventional IE, such as gastrointestinal complications, inconsistent image quality, and operator fatigue. The ROB'E RCR system for TEE has the potential to streamline and standardize TEE examinations, transform the landscape of IE, and expand access to life-changing treatments for patients with structural heart disease.

Future research should focus on refining the design of the RCR system, improving its user interface and safety protocols, and optimizing its performance in a wider range of clinical scenarios. Furthermore, it will be necessary to conduct clinical trials to assess the safety and efficacy of the system in humans.

## Ethics Statement

This study does not involve any experiments on human or animal subjects. As such, there were no ethical concerns or approvals required for this research. The development of the RCR for TEE focused solely on the design and technical aspects, without involving clinical trials or testing on living subjects.

## Funding

The authors have no funding to report.

## Disclosure Statement

D. Schewel and J. Schewel are co-founders of ROB'E GmbH, a company developing a robotic device for transesophageal echocardiography. The other authors have no conflicts to declare.

## Figures and Tables

**Suppl_Figure 4:**